# Screening Potential Reference Genes in *Tuta absoluta* with Real-Time Quantitative PCR Analysis under Different Experimental Conditions

**DOI:** 10.3390/genes12081253

**Published:** 2021-08-17

**Authors:** An-Pei Yang, Yu-Sheng Wang, Cong Huang, Zhi-Chuang Lv, Wan-Xue Liu, Si-Yan Bi, Fang-Hao Wan, Qiang Wu, Gui-Fen Zhang

**Affiliations:** 1 State Key Laboratory for Biology of Plant Diseases and Insect Pests, Institute of Plant Protection, Chinese Academy of Agricultural Sciences, Beijing 100193, China; yap2002@126.com (A.-P.Y.); yushengwang01@163.com (Y.-S.W.); lvzhichuang@caas.cn (Z.-C.L.); liuwanxue@caas.cn (W.-X.L.); bisiyan91@163.com (S.-Y.B.); wanfanghao@caas.cn (F.-H.W.); wuqiang@caas.cn (Q.W.); 2 Institute of Plant Protection, Xinjiang Academy of Agricultural Science, Urumqi 830091, China; 3 Guangdong Laboratory for Lingnan Modern Agriculture, Genome Analysis Laboratory of the Ministry of Agriculture, Agricultural Genomics Institute at Shenzhen, Chinese Academy of Agricultural Sciences, Shenzhen 518120, China; huangcong@caas.cn

**Keywords:** biological factors, environmental factors, expression stability, reference genes, RT-qPCR, South American tomato leafminer

## Abstract

*Tuta absoluta* is one of the most significant invasive pests affecting tomato plants worldwide. RT-qPCR has emerged as one of the most sensitive and accurate methods for detecting gene expression data. The screening of stable internal reference genes is the most critical step for studying the molecular mechanisms of environmental adaptability. The stable reference genes expressed in *T. absoluta* under specific experimental conditions have not yet been clarified. In this study, seven candidate reference genes (*RPL27*, *RPS13*, *RPS15*, *EF1-α*, *TUB*, *TBP*, and *β-actin*) and their optimal numbers were evaluated under biotic (developmental stages and adult tissues) and abiotic (insecticide, temperature, and plant VOC) conditions using four software programs. Our results identified the following reference genes and numbers as optimal: three genes (*EF1-α*, *RPS13*, and *RPL27*) for different developmental stages (egg, larva, pupa, unmated adult), two genes (*RPS13* and *TBP*) for adult tissues (antenna, head, thorax, abdomen, leg), two genes (*TBP* and *RPS13*) for insecticides (*Bacillus thuringiensis*, chlorpyrifos, abamectin-aminomethyl, and chlorantraniliprole), two genes (*RPL27* and *TUB*) for temperature-induced stresses (0, 25, and 40 °C), and two genes (*RPS13* and *TUB*) for VOC-induced stresses (nonanal, α-phellandrene, and tomato leaves). Our results provide a reference for selecting appropriate reference genes for further study of the functional genes of *T. absoluta* under different experimental conditions.

## 1. Introduction

Real-time quantitative polymerase chain reaction (RT-qPCR) is one of the most sensitive, convenient, and widely used methods for detecting gene expression in different biological samples [1,2,3]. It is also the most reliable method for detecting and quantifying transcriptional abundance [4]. When using RT-qPCR to study the expression levels of target genes, appropriate reference genes must be used to calibrate and standardize the experimental samples [5,6,7]. The reference genes should not be affected by external environments and should be stably expressed in various tissues or cells and under various experimental conditions [8]. However, many studies have demonstrated that the stability of different reference genes can change in response to different experimental conditions [9,10]. Using different normalization reference genes can lead to significant errors due to differences in sampling methods, total RNA extraction, efficiency of complementary DNA (cDNA) synthesis, transcriptional activity in biological samples, primer design, and reverse transcription efficiency, with some experimental differences estimated to be as high as 20- to 100-fold [11,12,13,14]. Therefore, it is important to identify and evaluate reference genes that are stably expressed under different experimental conditions to obtain more accurate and reliable experimental results [15].

Several genes have been confirmed as stable reference genes and are widely used when studying insects, such as *glyceraldehyde 3-phosphate dehydrogenase* (*GAPDH*), *actin* (*ACT*), *translation elongation factor 1α *(*TEF-1α*), *phospholipase A2* (*PLA2*), *arginine kinase* (*AK*), *tubulin* (*TUB*), *TATA binding protein* (*TBP*), and ribosomal proteins [7,16,17,18,19,20]. However, few reference genes are consistently expressed across all conditions (e.g., tissues, developmental stages, and experimental conditions) [19,21,22,23]. The screening of reference genes has recently been reported in many Lepidopteran insects, including *Plutella xylostella* [24,25], *Spodoptera litura* [22,26], *Chilo suppressalis* [27], *Helicoverpa armigera* [28], *Spodoptera exigua* [29], *Sesamia inferens* [30], *Bicyclus anynana* [19], *Danaus plexippus* [31], *Trichoplusia ni* [32], and *Lymantria dispar* [23]. However, reference genes can be expressed differently in different insect species or under different experimental conditions.

The South American tomato leafminer, also known as *T.absoluta* (Meyrick) (Lepidoptera: Gelechiidae), is one of the most important invasive insect pests globally and has spread to more than 80 countries and regions [33,34]. It has recently invaded China [35] and spread rapidly, becoming well-established in Xinjiang and Yunnan [36] and threatening the growth and development of local tomato plants [33,37,38]. Previous studies assessing *T. absoluta* have primarily focused on biological characteristics, insecticide resistance, invasiveness and spread pathway, and integrative pest management [33,39]; however, few have assessed the molecular mechanism behind the environmental adaptability of *T. absoluta*. The screening of stable internal reference genes is the most critical step for studying the molecular mechanisms of environmental adaptability (e.g., temperature, host plant, insecticide).

The ideal reference gene is stably expressed under different conditions [31,40]; however, the stable expression of any reference gene is relative since the perfect reference gene does not exist [23,41]. Therefore, identifying stable reference genes in a specific species under different experimental conditions is important to ensure the accuracy of the expression data of the target genes [27]. In this study, the stability of seven candidate reference genes (*ribosomal protein L27* (*RPL27*), *ribosomal protein s13* (*RPS13*), *ribosomal protein s15* (*RPS15*), *elongation factor1-α* (*EF1-α*), *TATA binding protein* (*TBP*), *tubulin* (*TUB*), and *β-actin*), all of which are commonly used in other Lepidopteran insects, were evaluated under different environmental conditions and different developmental conditions of *T. absoluta*. These results will inform subsequent studies of *T. absoluta* based on gene expression level, including those assessing the olfactory mechanism, environmental adaptability, the mechanism of insecticide resistance, and management using RNAi technology.

## 2. Materials and Methods

### 2.1. Insects

The *T. absoluta* populations were grown in tomato plants under laboratory conditions at 27 ± 2 °C, 50 ± 10% RH, and 14:10 h (light/dark) in Yunnan. Healthy and active insect individuals were used for the experiments.

### 2.2. Treatments

Experimental treatments included variable *T. absoluta* conditions and environmental factors. Four different development stages (egg, larva, pupa, and adult) were used for the pest and five adult tissues (antenna, head, thorax, abdomen, and leg) were assessed. For environmental conditions, the insecticides (four insecticides), temperature (three temperature settings), and plant volatile organic compounds (VOCs) (two VOCs and tomato leaves) were assessed. The samples of development stages, temperature, insecticides, and plant VOC treatment were collected at 11:00 am. The samples of tissues were collected at 10:00 am every time.

Five independent biological replicates were performed for each sample treatment. Each sample was collected and kept in a 1.5 mL centrifuge tube, immediately frozen in liquid nitrogen, and stored at −80 °C for subsequent experiments.

#### 2.2.1. Developmental Stages

Four different developmental stages were included: egg, larva (first, second, third, and fourth instar), pupa (male and female), and adult (male and female). Individuals of *T. absoluta* were randomly selected from the same colony, including the eggs (100 individuals per biological replicate), the 1st instar larvae (30 individuals), the 2nd instar larvae (3 individuals), the 3rd instar larva (1 individual), the 4th instar larva (1 individual), male (1 individual) and female (1 individual) pupae, and unmated male (3 individuals) and female (3 individuals) adults. In total, 45 samples (9 development stages/statuses × 5 biological replicates) were collected.

#### 2.2.2. Adult Tissues

Male and female adults were sampled separately. The adults were dissected to obtain samples of five different tissues, including the antenna (200 pairs per biological replicate), head (without antennae, 50 individuals), thorax (without wings and legs, 20 individuals), abdomen (10 individuals), and leg (50 individuals). Five types of different adult tissues were collected and were separately placed in 1.5 mL centrifuge tubes. In total, 25 samples (5 tissues × 5 biological replicates) were collected.

#### 2.2.3. Insecticides

Healthy, one-day-old third-instar larvae were treated with four common insecticides, specifically one microbial formulation BT (G033A) (provided by the Department of Biological Insecticide Research, IPP, CAAS, Beijing, China) and three chemical insecticides: chlorpyrifos (99.0%), abamectin-aminomethyl (98.5%), and chlorantraniliprole (97.2%) (Beijing Qincheng Yixin Technology Co., Ltd., Beijing, China). First, the chlorpyrifos and abamectin-aminomethyl were dissolved in acetone (*w*/*v*, 1:25) and chlorantraniliprole in dimethyl sulfoxide (DMSO) (1:250). Based on the results of previous studies [42,43,44], the concentration multiples of the four insecticides in this study were slightly changed. The concentrations of the three chemical insecticides were then diluted into 28.34 g/L, 0.5 mg/L, and 1.4 mg/L samples using distilled water containing 0.1% Triton X-100. The BT was diluted to a concentration of 1 g/L using distilled water containing 0.1% Triton X-100. The tomato leaves were each dipped into four insecticide solutions for 10 s and air-dried. The third instar larvae were starved for 12 h and individually fed with the dipped tomato leaves for 36 (BT) or 24 h (chlorpyrifos, abamectin-aminomethyl, and chlorantraniliprole), while distilled water containing 0.1% Triton X-100 was used as the control. In total, 20 samples/individuals (4 insecticides × 5 biological replicates) were collected.

#### 2.2.4. Temperatures

Three temperature treatments were designed based on previous studies, including cold-shock temperature treatment (0 °C) [45], heat-shock temperature treatment (40 °C) [46], and moderate temperature treatment (25 °C) [46]. The living, one-day-old third-instar larvae were treated for 1 h at all three setting temperatures. Five larval individuals were separately placed in five glass tubes to serve as five replicates, after which they were placed at setting temperatures for 1 h. For the 0 °C treatment, a heating refrigeration type circulator (CC-K6-NR, Peter Huber Kältemaschinenbau GmbH, Offengurg, Germany) was used; for 40 °C, a thermostat water bath (HH.S11-Ni2, Beijing Changan Scientific Instrument Factory) was used; and for 25 °C (as the control), a constant-temperature incubator (KRQ-250A, Shanghai Qixin Scientific Instrument Co., Ltd., Shanghai, China) was used. In total, 15 samples (3 temperatures × 5 biological replicates) were collected.

#### 2.2.5. Plant Volatile Organic Compounds (VOCs)

We used two kinds of plant VOCs: nonanal (96%, Beijing Bingda Biotechnology Co., Ltd., Beijing, China) and α-phellandrene (85%, Sigma, and tomato leaf (mature leaf) (*Solanum lycopersicum* cv. Shouhefenguan). The plant VOCs were diluted in hexane (5%, *v*/*v*), while the tomato leaflets (about 20 mm × 30 mm) were directly used as a control. Fifty newly emerged (one-day-old) male and female adults (sex ratio, 1:1) were placed in a 50 mL centrifuge tube and considered one biological replicate. Two hours later, one piece of filter paper (20 mm × 30 mm) was dripped with 20 μL of diluted plant VOC (nonanal or α-phellandrene) or a tomato leaflet, which was placed in the above centrifuge tube to excite the adults [47,48]. Five hours later, the heads of the excited adults were dissected and placed in a 1.5 mL centrifuge tube. In total, 15 samples (3 VOCs/tomato leaf × 5 biological replicates) were collected.

### 2.3. Total RNA Extraction and cDNA Synthesis

Total RNA was extracted using Trizol reagent (Invitrogen, Carlsbad, CA, USA), according to the manufacturer’s instructions. To reduce deviation, the purity and concentration of each RNA sample were determined twice using a NanoPhotometer^TM^ p330 (Implen, Munich, Germany). The A_260_/A_280_ ratios of all the RNA samples ranged from 1.8 to 2.2. The RNA integrity was tested using 1% agarose gel electrophoresis. Total RNA was used to synthesize cDNA (1 μg) using the *TransScript* All-In-One First-Strand cDNA Synthesis SuperMix for qPCR and PCR (Transgen Biotech, Beijing, China) according to the manufacturer’s instructions. The cDNA of each sample treatment had five biological replicates and was stored at −80 °C.

### 2.4. Candidate Reference Gene Selection and Specific Primer Design

Seven candidate reference genes (i.e., RPL27, RPS13, RPS15, EF1-α, TUB, TBP, and β-actin) commonly used in other Lepidoptera were selected based on the transcriptome data of *T. absoluta* (GeneBank accessions: MZ357900–357906). Primer pairs for each candidate reference gene were designed using the Primer Premier 5.0 software (Premier Biosoft International, Palo Alto, CA, USA), and the target candidate reference genes were amplified with optimal parameters (Table 1). The seven amplified reference genes were then amplified, sequenced (Sangon Biotechnology Co., Ltd., Shanghai, China), and validated. The specific RT-qPCR primer pairs targeted to *T. absoluta* were designed (Beacon designer 8.0 software (BioRad, Hercules, CA, USA) based on the validated sequences (Table 2). All primers were synthesized by Sangon Biotechnology Co., Ltd. (Shanghai, China).

### 2.5. Standard Curve Construction and RT-qPCR

Relative standard curves for the transcripts were generated with 2-fold serial dilutions of cDNA (1/2, 1/4, 1/8, 1/16, 1/32, and 1/64) extracted from the second instar larvae of *T. absoluta*. The corresponding RT-qPCR efficiencies (E) were determined for each gene and calculated according to the following equation: E = (10^[−1/slope]^ − 1) × 100 [49]. RT-qPCR was performed on the ABI 7500 PCR Detection System (Applied Biosystems, Waltham, MA, USA). The RT-qPCR reactions consisted of 10 µL Hieff^®^ qPCR SYBR^®^ Green Master Mix (Low Rox) (Yeasen Biotech Co., Ltd., Shanghai, China), 0.4 µL each forward and reverse primers (10 µM), and 1 µL the cDNA template. Thermal cycling conditions were as follows: an initial cycle at 95 °C (30 s), followed by 40 cycles of 10 s at 95 °C and 30 s at 60 °C. After all reactions, a melting curve analysis from 65 to 95 °C was used to ensure the consistency and specificity of the amplified product. According to the results of the standard curve, the cDNA template of all samples was diluted 5 times in the reference gene expression experiment. Each treatment included five biological replicates, and each reaction was performed in triplicate.

### 2.6. Statistical Analysis

Relative cycle threshold (C_t_) values are widely used to identify stably expressed genes by RT-qPCR analysis. The average C_t_ value was calculated based on five biological replicates. Two commonly used software tools, geNorm version 3.5 [13] and NormFinder version 0.953 [50], were used to evaluate the raw average C_t_ values of the seven selected reference genes, according to the manufacturer’s instructions. For both geNorm and NormFinder, the 2^−ΔCt^ value (ΔC_t_ = the corresponding C_t_ value—minimum C_t_ value) was used to calculate the stability value of the reference genes. The values of the expression stability measure (M value) and the pair-wise variation (V) of the target gene were evaluated using the geNorm software. An M value below 1.5 indicates that the gene can be used as a reference gene, while the gene with the lowest M value should be the most stable [13]. When V*_n_*_/*n*+1_ is below 0.15, it indicates that the most suitable number of reference genes for normalization is *n*; when the V*_n_*_/*n*+1_ is above 0.15, it indicates that the most suitable number is *n*+1 [13]. The stability value (SV) was evaluated by the NormFinder software and was used to rank the expression stability of the suitable reference genes. The gene with the lowest value is typically the most stable. The standard deviation (SD) was calculated according to the average C_t_ value using BestKeeper [51]. The lower the standard deviation, the better the stability of the reference gene. However, if the SD > 1, this indicates that the expression of the reference gene is unstable. Finally, the comprehensive and user-friendly web-based comprehensive tool RefFinder (https://localhost/RefFinder-master/RefFinder-master/?type=reference (accessed on 15 March 2021)) [52] was used to calculate the geometric mean (GM) values according to the results of these algorithms, after which the candidate reference genes were ranked based on GM. The most suitable reference genes under each condition/treatment were identified according to the GM value and the number of optimal reference genes determined by the V value. The difference of gene expression was analyzed by post-hoc testing using IBM SPSS statistics 25.0 (SPSS Inc., Chicago, IL, USA) with a threshold of *p* < 0.05.

## 3. Results

### 3.1. Acquisition of Candidate Reference Gene Sequences

The sequences of the seven candidate genes, namely *RPL27*, *RPS13*, *RPS15*, *EF1-α*, *TUB*, *TBP*, and *β-actin*, were obtained using the transcriptome database of the antenna of *T. absoluta* (unpublished data). The sequences were validated by PCR (Figure S1) and then sequenced and confirmed to exhibit 100% identity with the corresponding transcriptome sequence data (Table 1).

### 3.2. Specificity and Amplification Efficiency of Specific RT-qPCR Primers

Based on the validated sequences of the seven reference genes, we designed the specific RT-qPCR primers (Table 2). The accuracy of the RT-qPCR primers was confirmed by the presence of a single peak in the melting curve analyses (Figure S2) and a single amplicon on 1% agarose gel. The sizes of the amplified products were consistent with the expected sizes, indicating that each primer pair was specific. The amplification efficiency of each primer pair under RT-qPCR analysis for the seven candidate reference genes ranged from 92.7 to 110.2%, while the linear regression coefficient *R*^2^ ranged from 0.99 to 1.00 based on the standard curve (Table 2). This demonstrated that all seven primer pairs met the requirements of RT-qPCR.

### 3.3. Expression Levels of the Candidate Reference Genes

**Figure 1:**
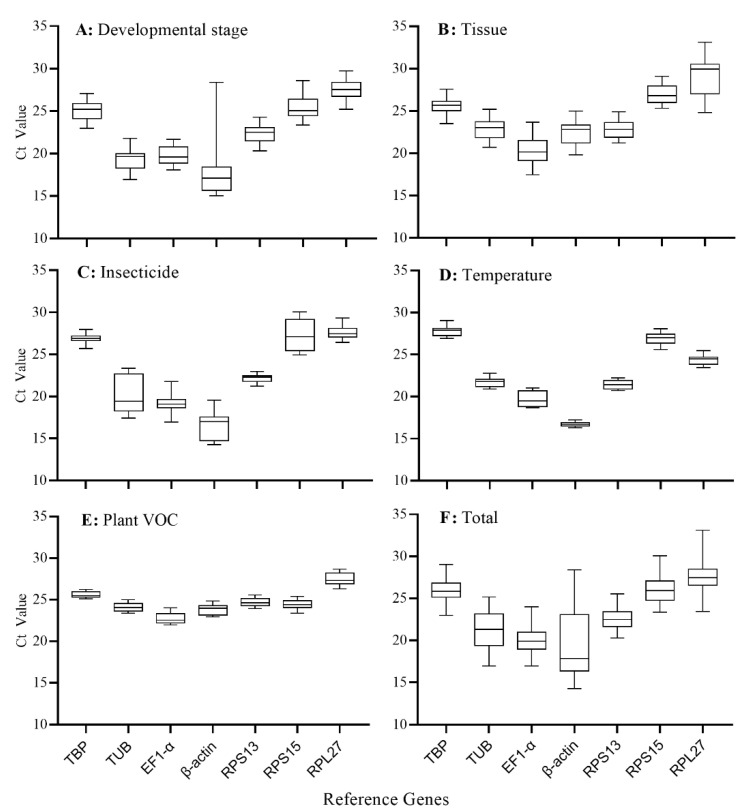
Expression levels of seven candidate reference genes in *T. absoluta*. Line across the box shows the median value, while inside the box indicates Ct values. The top and bottom whiskers represent the 25th and 75th percentages, respectively. Developmental stages (**A**), adult tissues (**B**), insecticides (**C**), temperatures (**D**), plant VOCs (**E**), and total (**F**) based on C_t_ values of all samples exhibited in the five experimental conditions.

The transcription levels of the seven candidate reference genes were evaluated using the C_t_ values obtained from the RT-qPCR analysis. The C_t_ values of the seven candidate reference genes varied under the five experimental conditions (Table 3). Under the different developmental stages, *β-actin* (C_t_ = 18.11) showed the highest expression level, and *RPL27* (C_t_ = 27.49) showed the lowest expression level, but the gap between the maximum (C_t_ = 28.39) and the minimum values (C_t_ = 15.03) of *β-actin* was the largest (Figure 1A). This could be related to the expression of the *β-actin* in the eggs (C_t_ = 27.43), which was significantly lower than in other developmental stages (C_t_ = 16.85) (data not shown). In different adult tissues, the expression level of *EF1-α* (C_t_ = 20.30) was the highest, while *RPL27* (C_t_ = 28.88) was the lowest (Figure 1B). For the insecticide-induced stresses, the expression level of *β-actin* (C_t_ = 16.33) was the highest, and the expression level of *RPL27* (C_t_ = 27.63) was the lowest (Figure 1C). For the expression levels of all samples of temperature-induced stresses, *β-actin* was the highest (C_t_ = 16.71), and *TBP* was the lowest (C_t_ = 27.80) (Figure 1D). Under plant VOC exposure conditions, the expression level of *EF1-α* (C_t_ = 22.74) was the highest, and the expression level of *RPL27* (C_t_ = 27.45) was the lowest (Figure 1E). For the total C_t_ values of the five experimental conditions, the lowest C_t_ value was detected in *β-actin* (C_t_ = 14.26), and the highest was in *RPL27* (C_t_ = 33.11). The C_t_ value of *β-actin* varied most due to differences between the eggs and other treatments. The expression levels of both *β-actin* and *EF1-α* were the highest, with average C_t_ values of 19.36 and 20.19, respectively. The lowest expression level was *RPL27*, with an average C_t_ value of 27.47 (Table 3, Figure 1F).

### 3.4. Expression Stability and Ranking of the Candidate Reference Genes

#### 3.4.1. Developmental Stages

**Figure 2:**
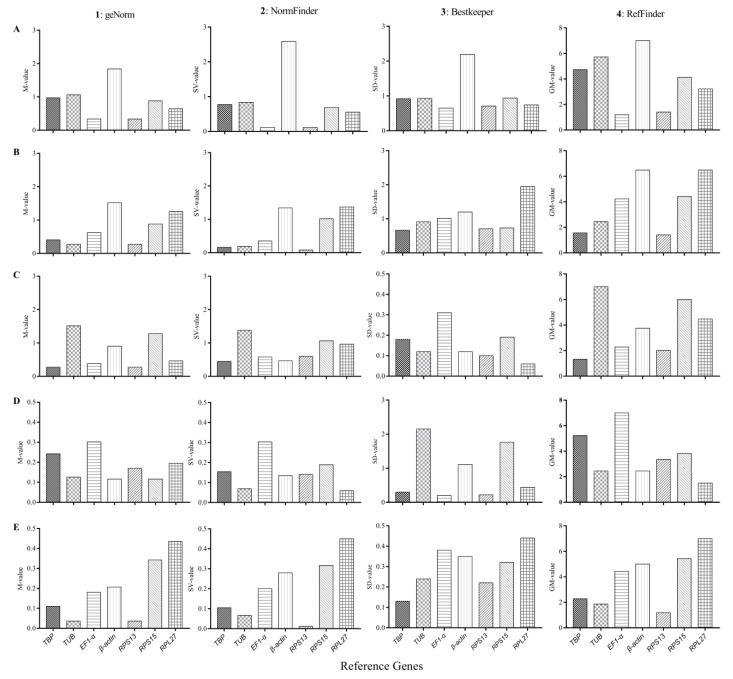
Stability analysis of the seven candidate reference genes expression of *T. absoluta* under the following experimental conditions: developmental stage (**A**), adult tissue (**B**), insecticide (**C**), temperature (**D**), and plant VOC (**E**), as calculated by four different software programs: geNorm (**1**), NormFinder (**2**), BestKeeper (**3**), and RefFinder (**4**).

**Figure 3:**
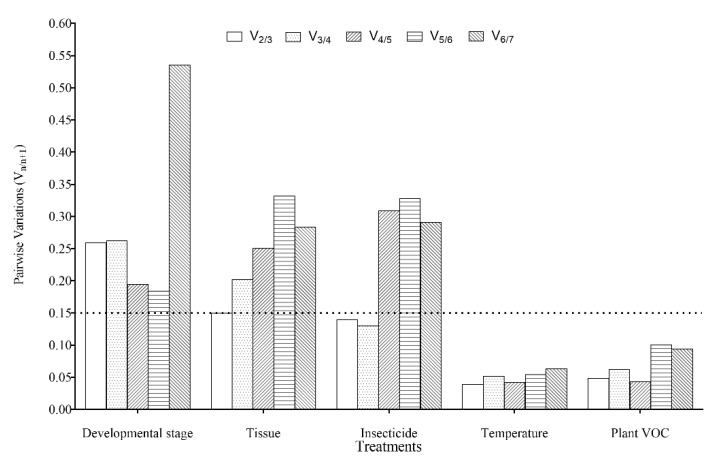
Optimal number of reference genes for normalization in RT-qPCR of *T. absoluta* under different conditions. The pairwise variation (V*_n_*/V*_n_*_+1,_ *n* is the number of reference genes) analyzed using the geNorm software was used to determine the optimal number of reference genes required for accurate normalization. Black dotted lines indicate the threshold for paired variation values (V*_n_*/V*_n_*_+1_ = 0.15). V*_n_*_/*n*+1_ < 0.15 indicates that the optimal number of reference genes for normalization is *n*, and the V*_n_*_/*n*+1_ > 0.15 indicates that the optimal number is *n*+1.

Analysis using the geNorm software determined that the M values of *β-actin* exceeded 1.5, indicating that *β-actin* could not be used as the stable reference gene in this treatment (Figure 2(A1)). Both *EF1-α* and *RPS13* were the most stable reference genes as indicated by the geNorm, NormFinder, and BestKeeper software programs (Figure 2(A1–A3)). RefFinder was used to comprehensively rank the highest to the lowest stability: *EF1-α*, *RPS13*, *RPL27*, *RPS15, TBP*, *TUB*, and *β-actin* (Figure 2(A4)). The pairwise variation value V2/3 (0.259) exceeded 0.15, according to analysis using geNorm software (Figure 3), suggesting that the optimal number of reference genes for normalization is three genes at different developmental stages: *EF1-α*, *RPS13*, and *RPL27* (Figure 2(A3,A4)).

#### 3.4.2. Adult Tissues

Based on analysis using the geNorm software, *TUB* and *RPS13* were the most stable reference genes (Figure 2(B1)). *RPS13* and *RPL27* were the most and least stable reference genes, respectively, based on analysis using the NormFinder software (Figure 2(B2)). Based on analysis using the BestKeeper program, *TBP* was the most stable reference gene, and *RPL27* was the least stable reference gene (Figure 2(B3)). Based on analysis using the RefFinder program, the reference genes ranked from the most to least stable were: *RPS13*, *TBP*, *TUB*, *EF1-α*, *RPS15*, *β-actin*, and *RPL27* (Figure 2(B4)). Analysis using the geNorm program indicated that the pairwise variation value V2/3 (0.149) was below 0.15, meaning that both *RPS13* and *TBP* could be used for normalization in the different adult tissues of *T. absoluta* (Figure 2(B4) and Figure 3).

#### 3.4.3. Insecticide-Induced Stress

*TBP* and *RPS13* were the most stable reference genes, based on analysis using the geNorm software (Figure 2(C1)). *TBP* was the most stable reference gene, based on analysis using the NormFinder software (Figure 2(C2)). *EF1-α* and *RPS13* were the most stable reference genes, based on analysis using the BestKeeper software (Figure 2(C3)). *TUB* was the most unstable reference gene according to all three programs (Figure 2(C1–C3)). Based on analysis using the RefFinder software (Figure 2(C4)), the reference genomes ranked from most to least stable were *TBP*, *RPS13*, *EF1-α*, *β-actin*, *RPL27*, *RPS15*, and *TUB*. Both *TBP* and *RPS13* could be selected for normalization under insecticide-induced stresses of *T. absoluta*, based on analysis using the RefFinder and geNorm programs (Figure 2(C4) and Figure 3).

#### 3.4.4. Temperature-Induced Stress

Based on analysis using the geNorm software, the reference genes with the most stable expression were *β-actin* and *RPS15*, while *EF1-α* was the most unstable reference gene (Figure 2(D1)). *RPL27* was the most stable reference gene, and *EF1-α* was the least stable gene, based on analysis using the NormFinder and BestKeeper software (Figure 2(D2,D3)). Based on analysis using the RefFinder software, the reference genomes ranked from most to least stable were *RPL27*, *TUB*, *β-actin*, *RPS13*, *RPS15*, *TBP*, and *EF1-α* (Figure 2(D4)). Both *RPL27* and *TUB* could be selected for normalization under cold- and heat-shock treatments for *T. absoluta*, based on analysis using the RefFinder and geNorm programs (Figure 2(D4) and Figure 3).

#### 3.4.5. Plant VOC-Induced Stress

Both *TUB* and *RPS13* were the most stable reference genes based on analysis using the geNorm and NormFinder programs (Figure 2(E1,E2)). *TBP* was the most stable reference gene based on analysis using the BestKeeper software (Figure 2(E3)). However, *RPL27* was the least stable reference gene based on analysis using the geNorm, NormFinder, and BestKeeper programs (Figure 2(E1–E3)). Based on analysis using the RefFinder software, the reference genomes ranked from most to least stable were *RPS13*, *TUB*, *EF1-α*, *TBP*, *β-actin*, *RPS15*, and *RPL27* (Figure 2(E4)). Both RPS13 and TUB could be selected and used for normalization under the condition of plant VOC exposure of *T. absoluta*, based on analysis using the geNorm and RefFinder programs (Figure 2(E4) and Figure 3).

## 4. Discussion

Quantitative real-time polymerase chain reaction is an important technique for analyzing gene expression in various biological systems [2,3,53] and has become the standard technique for detecting mRNA levels in gene expression. RT-qPCR uses a relative quantitative method to evaluate the expression level of the target gene based on a stable reference gene and is both highly sensitive and highly accurate [5]. The stability of the reference genes directly affects the accuracy of the expression of a target gene. The reference genes (housekeeping genes) are expressed in all kinds of cells and play an important role in maintaining the basic life activities of cells. The expression of reference genes varies greatly in different types of cells, tissues, and under various experimental conditions [11,12,13]. Therefore, selecting suitable and stable reference genes is a crucial first step to study gene expression and ensure the reliability of gene expression data.

The stability value and rank of each reference gene were all evaluated with three commonly used software programs: BestKeeper, geNorm, and NormFinder. However, the stability ranks of the analyzed genes were inconsistent due to different algorithms used by each of these analysis tools, an anomaly that has been identified in previous studies [22,26,28]. Therefore, the comprehensive ranking of the stability of the reference genes was evaluated using the RefFinder software. Furthermore, selecting more than one reference gene at the same time, under certain conditions, can improve both the accuracy and reliability of the resulting data [26]. Therefore, the appropriate number of reference genes should be determined according to the pair mutation value (V*_n_*_/*n*+1_) using the geNorm software. If the V*_n_*_/*n*+1_ is less than 0.15, the optimal number of reference genes is *n*; if the V*_n_*_/*n*+1_ is greater than 0.15, the optimal number of reference genes is *n*+1. In this study, it is most appropriate to select at least two reference genes under specific experimental conditions.

The classical reference genes, including *ACT*, *TBP*, *TUB*, *EF1-α*, and ribosomal proteins, are widely expressed in organisms and are typically used in data normalization. Ribosomal proteins are the primary components of ribosomes and play an important role in protein biosynthesis in cells [54,55]. *Elongation factors (EFs)* promote polypeptide chain elongation during mRNA translation and are highly conserved in organisms [56]. *TUB* and *ACT* are skeleton proteins. These are often used as reference genes [57]. However, these reference genes are not consistently expressed under all conditions [26,29], so the stability of the reference genes should be evaluated under specific conditions.

In this study, the expression stability of seven candidate reference genes (*RPL27*, *RPS13*, *RPS15*, *EF1-α*, *TUB*, *TBP*, and *β-actin*) were evaluated with various biological factors (development stages, adult tissues) and environmental factors (insecticides, temperatures, plant VOCs). To our knowledge, this is the first study to evaluate the expression stability of the reference genes used in *T. absoluta*. Our results demonstrate that the most suitable candidate combinations of reference genes were *EF1-α*, *RPS13*, and *RPL27* for different developmental stages; *RPS13* and *TBP* for adult tissues; *TBP* and *RPS13* for insecticide-induced stresses; *RPL27* and *TUB* for temperature-induced stresses; and *RPS13* and *TUB* for plant VOC exposure. No reference genes were stably expressed under all experimental conditions. For example, *TBP* was only stable for adult tissues and insecticide; *TUB* was stable for plant VOC exposure and temperature treatments but unstable for insecticide-induced stresses; *RPL27* was stable for temperature stresses and developmental stages but unstable for adult tissues and plant VOC exposure; and *EF1-α* was stable for developmental stages but unstable for temperature-induced stresses. Our results align with those obtained in previous studies. *EF1-α* is the most stable gene for the different developmental stages of *S. inferens* [30], *P. xylostella* [24], *S. exigua* [29], and *C. suppressalis* [27]; *β-actin* is the least stable gene in *L. dispar* [23]. For different insect tissues, *RPS13* is the optimal reference gene for *S. inferens* [30]. *RPL27* is the optimal reference gene under temperature-induced stresses in *H. armigera* [28].

However, different insect species had different results: *β-actin* is the most stable reference gene for the developmental stages of *Phyllonorycter ringoniel* [58], *P. xylostella* [24], *S. exigua* [29], *Mythimna separata* [59], and *S. inferens* [30]; TUB is the most stable reference gene for *L. dispar* [23]. *EF1* is the most stable reference gene for different insect tissues of *S. litura* [22], *P. xylostella* [24], and *S. inferens* [30]; and *ACT* is the most stable reference gene for *C. suppressalis* [27]. *β-actin* is the most stable reference gene for insecticide-induced stresses on *S. litura* (insecticides: chlorpyrifos, diafenthiuron, spinosad, indoxacarb, or chlorantraniliprole, treated for 48 h) [22]; *RPS15* is the most stable reference gene for *H. armigera* (BT, β-cypermethrin, and Spinetoram; treated for 24 and 48 h, respectively) [28]. *RPS15* is the most stable reference gene for temperature-induced stresses of *H. armigera* (T = 4, 27, and 40 °C; treated for 2, 6, and 12 h, respectively) [28]. These differences could be caused by differences across species (such as development stages and adult tissues) and/or differences in treatment conditions (such as insecticides and temperatures). In the case of plant VOCs, Liu used *β-actin* as a reference gene under plant VOC treatment in *Diaphorina citri* [48].

In this study, we assessed seven stably selected reference genes (*RPL27*, *RPS13*, *RPS15*, *EF1-α*, *TUB*, *TBP*, and *β-actin*) in *T. absoluta* under different biotic factors (development stage and adult tissue) and abiotic factors (insecticide, temperature, and plant VOC) to normalize the expression of the target genes during RT-qPCR analysis. Our results demonstrated that the stability of the reference genes was inconsistent under different experimental conditions. No single gene was stably expressed across different developmental stages, adult tissues, and experimental conditions [21], and the stability of the reference genes varied among different insect species. Therefore, multiple reference genes should be used to obtain reliable and accurate results in quantitative expression studies of functional genes when study gene expression using RT-qPCR analysis.

## 5. Conclusions

This experiment was the first in-depth study to identify and validate reference genes for RT-qPCR data normalization under different biotic and abiotic stresses in *T. absoluta*. Our results indicate that *EF1-α*, *RPS13*, and *RPL27* had the greatest stability across various developmental stages, while *RPS13* and *TBP* had the greatest stability across various adult tissues of *T. absoluta*. *TBP* and *RPS13* were the most stable genes under insecticide treatments; *RPL27* and *TUB* had the greatest stability under thermal (heat- and cold-shock) treatments; and *RPS13* and *TUB* had the greatest stability under plant VOC treatments. Based on all the experimental conditions, *RPS13* can be used under all conditions except temperature treatment, while *RPL27* and *TUB* can be used as supplements under temperature treatment. The expression stability of a putative reference gene is typically verified prior to each RT-qPCR experiment by setting the experimental conditions. To obtain reliable and accurate results, multiple reference genes should be used when analyzing quantitative gene expression. Our results provide comprehensive information on the reference genes needed to study gene expression in *T. absoluta* and other invasive Lepidopteran pests.

## Figures and Tables

**Table 1:** Candidate reference genes and primers used in this study.

Gene Name	Primer	Primer Sequences (5′-3′)	Tm (°C)	Amplicon Length (bp)
*TUB*	TUB-F TUB-R	GAGTGCATCTCAGTCCACGTT GAAGGAGTGGAAGATGAGGAAT	55	417
*β-actin*	β-actin-F β-actin-R	TCCTTGGAGATCCACATCTG CCCATCTACGAAGGTTACG	53	590
*EF1-α*	EF1-α-F EF1-α-R	GCCCTTCTCGCCTTCACCCTT GGGCACCGTTCCAATACCACC	62	381
*RPL27*	RPL27-F RPL27-R	GTAAGATTATGAAACCTGGGAA ACCTAAGCTTTTGGAAGAACC	51	395
*RPS13*	RPS13-F RPS13-R	CCCTACCTGGTTGAAACTGAC TGAAGCGGTGCTAGACTCATA	51	376
*TBP*	TBP-F TBP-R	TCCTCACCACTTGTCGGCT GTTGTCCGTGGCTCTCTGAT	58	374
*RPS15*	RPS15-F RPS15-R	CGAGAAATACCCCAGAAATGT TTCTTCGCTTGTTGTTTGATT	51	429

**Table 2:** Reference genes and specific primers used for RT-qPCR analysis in *T. absoluta*.

Primer	Primer Sequences (5′-3′, F/R)	Amplicon Length (bp)	Tm (°C)	Efficiency (%)	R^2^
RPL27	CGTTACTCGGTAGACTTCA GTTCTTTCCACTCTTGTATCG	126	60	92.7	0.992
RPS13	AGTTGCCCAAGTCAGATT TTCCAAGTGCTTCCTCAT	133	60	102.0	0.999
RPS15	ATACGAAGTGAACAGTTAGC ACGGTCCATTCTTCCAAT	92	60	103.8	0.990
EF1-α	AGTCTCCTCATACATCAAGAAG CCTCCTTACGCTCAACAG	143	60	100.7	0.998
TBP	GTCTACTCCACACCTCAA CTGCCACTCGTTATCATTG	77	60	110.2	0.993
TUB	GGAGTCCAGATCGGTAAC GCTGAAGAAGGTGTTGAAC	126	60	95.9	0.998
β-actin	CAGGTCCTTACGGATGTC CTCTTCCAGCCTTCCTTC	99	60	104.4	0.996

**Table 3:** Distribution of the Ct values of each candidate reference gene in *T. absoluta* (mean ± SD).

Gene Name	Total Samples	Developmental Stage	Tissue	Insecticide		Temperature		Plant VOC	
				Treatment	Control	Treatment	Control	Treatment	Control
*TBP*	25.84 ± 1.28 b	25.14 ± 1.15 b	25.45 ± 0.98 b	26.92 ± 0.51 a	27.64 ± 0.52 a	27.80 ± 0.65 a	27.84 ± 0.64 a	25.61 ± 0.39 b	25.54 ± 0.35 b
*TUB*	21.14 ± 2.22 d	19.31 ± 1.27 d,e	22.81 ± 1.16 c	20.41 ± 2.29 b,c	21.34 ± 0.44 d	21.78 ± 0.60 d	21.63 ± 0.74 d	24.09 ± 0.51 c,d	23.88 ± 0.47 d
*EF1-α*	20.19 ± 1.50 d,e	19.80 ± 1.04 d	20.30 ± 1.47 d	19.22 ± 1.04 c	20.12 ± 0.83 e	19.74 ± 0.89 e	19.92 ± 0.85 e	22.74 ± 0.69 e	22.35 ± 0.37 e
*β-actin*	19.36 ± 3.68 e	18.11 ± 3.69 e	22.42 ± 1.41 c	16.33 ± 1.70 d	16.50 ± 0.15 f	16.71 ± 0.25 f	16.58 ± 0.21 f	23.83 ± 0.63 d	23.69 ± 0.59 d
*RPS13*	22.61 ± 1.22 c	22.30 ± 1.00 c	22.83 ± 0.95 c	22.16 ± 0.49 b	21.28 ± 0.46 d	21.44 ± 0.55 d	21.46 ± 0.58 d	24.72 ± 0.50 c	24.51 ± 0.41 c
*RPS15*	26.08 ± 1.62 b	25.35 ± 1.31 b	26.94 ± 1.14 b	27.39 ± 1.91 a	26.57 ± 0.53 b	26.93 ± 0.70 b	26.69 ± 0.53 b	24.44 ± 0.63 c,d	24.08 ± 0.41 c,d
*RPL27*	27.47 ± 1.84 a	27.49 ± 1.15 a	28.88 ± 2.34 a	27.63 ± 0.74 a	24.17 ± 0.57 c	24.37 ± 0.61 c	24.38 ± 0.69 c	27.45 ± 0.76 a	26.99 ± 0.42 a

Lowercase letters indicate the Ct value of each reference gene was significant different under the same treatment (*p* < 0.05, one-way ANOVA followed by LSD test).

## Data Availability

The data presented in this study are available in article.

## Supplementary Materials

The following are available online at https://www.mdpi.com/article/10.3390/genes12081253/s1, Figure S1: Single amplicon of desired size for the seven candidate reference genes in *T. absoluta* visualized on 1% agarose gel, Figure S2: Melting curves and melting peaks of the seven candidate reference genes in *T. absoluta*.
