# Supplementary material for: Screening Potential Reference Genes in Tuta absoluta with Real-Time Quantitative PCR Analysis under Different Experimental Conditions
Source: Genes (Basel). 2021 Aug 17;12(8):1253. doi: 10.3390/genes12081253 (PMC8391263; doi:10.3390/genes12081253)
Supplement: Supplementary file 1 [file genes-12-01253-s001.zip › genes-1287204-supplementary.pdf]

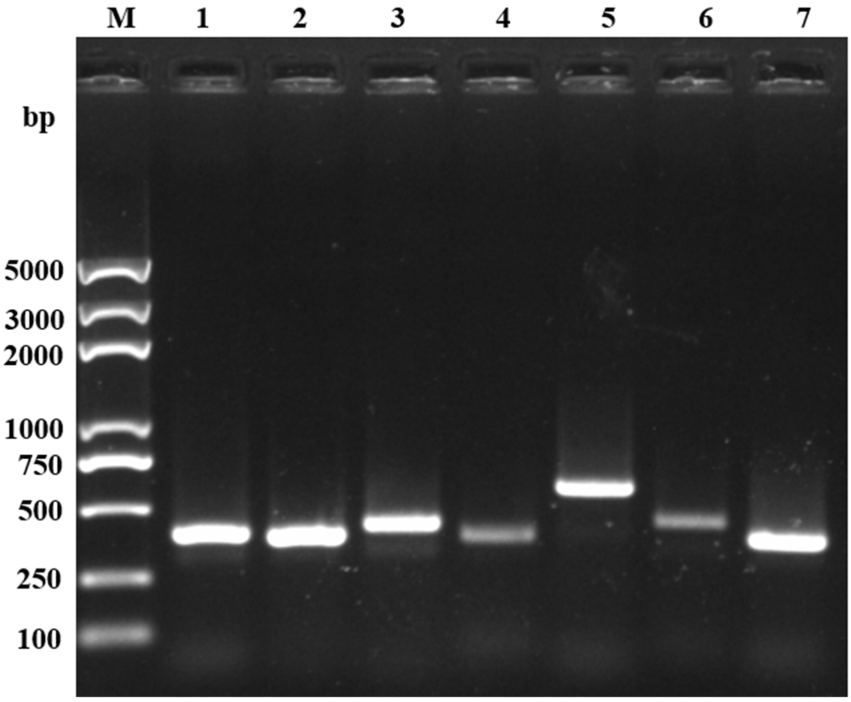

Figure S1. Single amplicon of desired size for the seven candidate reference genes in *Tuta absoluta* visualized on 1% agarose gel. DNA ladder marker (M), RPL27 (1), EF1-*α* (2), TUB (3), TBP (4), β-actin (5), RPS15 (6), and RPS13 (7) genes, respectively.

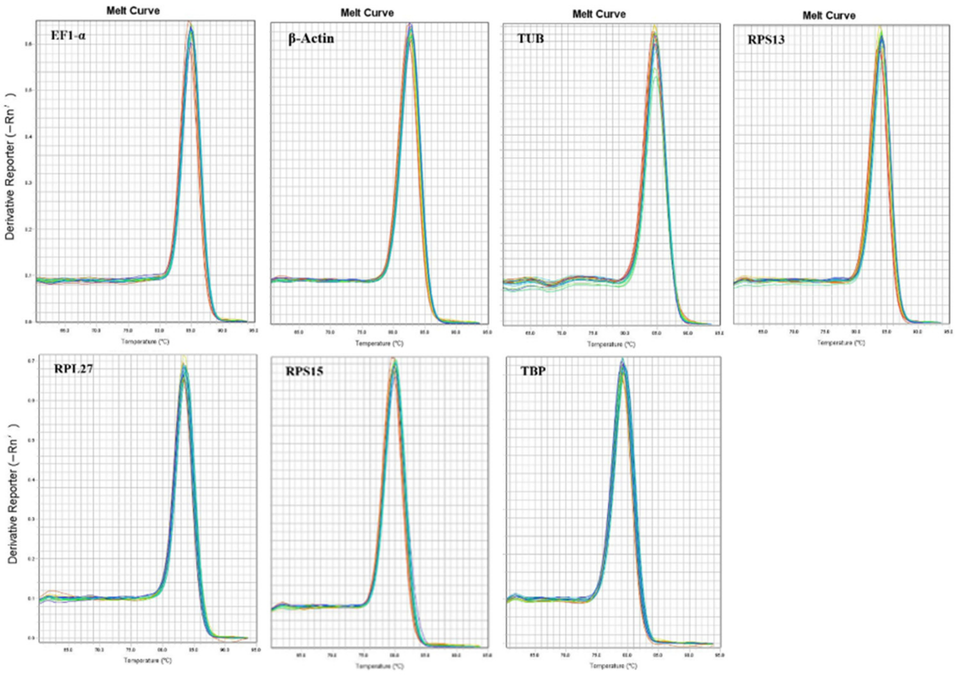

Figure S2. Melting curves and melting peaks of the seven candidate reference genes in *Tuta absoluta*.
